# Impact of Childhood Adversity and *Vasopressin receptor 1a* Variation on Social Interaction in Adulthood: A Cross-Sectional Study

**DOI:** 10.1371/journal.pone.0136436

**Published:** 2015-08-21

**Authors:** Jia Jia Liu, Fenglan Lou, Catharina Lavebratt, Yvonne Forsell

**Affiliations:** 1 School of Nursing, Shandong University, Jinan, 250012, China; 2 Neurogenetics Unit, Department of Molecular Medicine and Surgery, Karolinska Institutet, Stockholm, Sweden; 3 Center for Molecular Medicine, Karolinska University Hospital, Stockholm, Sweden; 4 Department of Public Health Sciences, Karolinska Institutet, Stockholm, Sweden; University of Rouen, France, FRANCE

## Abstract

**Background:**

Arginine vasopressin (AVP) plays a role in social behavior, through receptor AVPR1A. The promoter polymorphism *AVPR1A* RS3 has been associated with human social behaviors, and with acute response to stress. Here, the relationships between *AVPR1A* RS3, early-life stressors, and social interaction in adulthood were explored.

**Methods:**

Adult individuals from a Swedish population-based cohort (*n* = 1871) were assessed for self-reported availability of social integration and social attachment and for experience of childhood adversities. Their DNA samples were genotyped for the microsatellite *AVPR1A* RS3.

**Results:**

Among males, particularly those homozygous for the long alleles of *AVPR1A* RS3 were vulnerable to childhood adversity for their social attachment in adulthood. A similar vulnerability to childhood adversity among long allele carriers was found on adulthood social integration, but here both males and females were influenced.

**Limitation:**

Data were self-reported and childhood adversity data were retrospective.

**Conclusions:**

Early-life stress influenced the relationship between *AVPR1A* genetic variants and social interaction. For social attachment, *AVPR1A* was of importance in males only. The findings add to previous reports on higher acute vulnerability to stress in persons with long *AVPR1A* RS3 alleles and increased AVP levels.

## Introduction

Individuals’ functional social behavior depends on their capacity for social interaction, which plays an important role in having an active life and participating in society [1]. When facing stress or other health risk events, social interaction works as a buffer promoting adaptive behavior of neuroendocrine responses against negative health effects [2]. It is well known that environmental factors, especially in early life, play a critical role in the individual’s development, e.g. traumatic events experienced in early childhood may have a long-term harmful effects [3]. Large bodies of studies have revealed that experiences of childhood adversity are associated with increased risk of psychiatric disorders and disturbed social interaction in adulthood [4–6].

Arginine vasopressin (AVP) is widely expressed in the brain and has been shown to play an important role in social behavior, including social recognition, aggression, reproduction, parenting, affiliation, through comparative neurobiological studies conducted in species from nematodes to mammals [7–9]. AVP functions through activation of specific receptors, including V_1a_ (AVPR1A), V_1b_ (AVPR1B) and V_2_ (AVPR2). The V_2_ receptors are expressed essentially in kidneys and are responsible for the antidiuretic function, and are therefore not relevant to study in brain.

Following evidence from vole models, that the expression of *AVPR1A* gene in brain is responsible for intra-and inter-species variation in social behavior [10–13], genetic research focused on human social behavior has paid special attention to the *AVPR1A* gene. In human’s *AVPR1A* gene (chr 12q14-15), there are three microsatellite repeats, [GT]_25_, RS1, and RS3, located in the 5‘ flanking region, of which, RS3 has been most frequently studied. Studies have linked the length of these repeats to behavioral traits, suggesting that the repeats are relevant for brain function related to emotional arousal and social behavior [14].

The first molecular genetic studies of *AVPR1A* and human behavior was conducted in families with autism, characterized by impairment in social behavior, and demonstrated transmission disequilibrium between autism and both RS1 and RS3 [15,16]. In addition to studies involving autism, research in healthy individuals has, as stated above, linked these upstream promoter-region microsatellites of *AVPR1A* to human social behaviors. First, associations between both microsatellites (RS1 and RS3) and self-presentation and sibling social relationships was found [17]. In following studies, longer alleles of RS3 were found to be associated with higher level of economic altruism and greater level of prepulse inhibition, which is an indicator of social cognition [18,19]. In a recent study conducted in preschoolers, a specific RS3 allele (corresponding to the high frequency allele of 334 bp) was negatively associated with altruistic behavior [20]. This 334 bp ‘risk’ allele was found to associate also with poor pair-bonding behavior in men [21].

The mechanistic explanation to the associations between *AVPR1A* RS3 length and social behavior remains to be clarified, but there are reports suggesting a functionality of the RS3 polymorphism. *AVPR1A* RS3 alleles, longer than the risk allele, were associated with higher *AVPR1A* mRNA levels in human postmortem hippocampal samples [18], in accordance with the findings from voles in amygdala, thalamus and olfactory bulb [12]. Moreover, in human subjects, having the longer alleles of RS3 was associated with stronger amygdala activation in response to fearful faces [22].

In the present study, we aimed at exploring the relationships between *AVPR1A* RS3 variation, early stressors, i.e. experience of childhood adversity, and social interaction in adulthood. We hypothesized that longer RS3 alleles would associate with higher levels of social interaction and that exposure to childhood adversity would reduce the associations. We studied two measures of social interaction: social integration reflecting the number of friends available to interact with, and social attachment reflecting the availability to close social relationships with adults. These two concepts represent quantitative social interaction considering both distant contacts and close relationships, and they also reflect the individual’s capability to have close relationships [23].

## Materials and Methods

### Ethics statement

The PART study was approved by the ethical review board of Karolinska Institutet, and written informed consent was obtained from all participants. The investigation was carried out in accordance with the latest version of the Declaration of Helsinki.

### Participants

The study sample derived from the PART study, a longitudinal population-based study aimed at identifying risk and protective factors for mental health in Stockholm County, Sweden. Participants were randomly selected 20–64 years old Swedish nationals. Self-reported questionnaire data were collected in 1998–2000 (wave 1) and participants were followed-up 3 years later (wave 2) with a similar questionnaire. Of the participants 11% were born outside Sweden, the vast majority with Nordic origin. Details of the study and sampling procedures can be found elsewhere [24]. The questionnaire covered demographic characteristics, childhood conditions, social network, financial status, negative life events, use of drugs, somatic health, and psychiatric symptoms. A DNA collection was performed, with a nested case control design, of those participating in both wave 1 and wave 2 [25]. Of the DNA collected (*n* = 3018), a random subsample was selected for genotyping (*n* = 2200). In the subsample, 29% were cases i.e. 21% reported symptoms fulfilling a depression diagnosis (major depression, dysthymia or mixed-anxiety depression) and 8% an anxiety diagnosis, according to DSM-IV, in any wave. These rates are higher than the corresponding 12% and 4% among all the participants who responded to the questionnaires in both wave 1 and wave 2. Therefore, for those with depression or anxiety, data on childhood adversity and social interaction were used from the wave of diagnosis. Controls were those without a depression or anxiety diagnosis. For the controls, and those with a diagnosis in both waves, data were used from wave 1.

### Childhood adversity and social interaction variables

Childhood adversity (CA) before 18 years of age was assessed with three questions. “Did one of your parents die?” If the answer is no, then scored as 1; if yes, scored as 2. For the questions “Did your family have financial problems?” and “Did friction exist in your family?”, a three-point likert scale was used from 1 (no) to 3 (2 = yes, milder or shorter periods, 3 = yes, more difficult or longer periods). The items were summed up (CA) and used as a category variable both coded in two-levels (non-CA [score = 3] and CA [score>3]) and in three-levels (non-CA [score = 3], mild-moderate-CA [score = 4 or 5], and high-CA [score>5] group).

Four questions of Availability of social integration (AVSI) and 5 questions of Availability of attachment (AVAT) were used to assess social relationships. AVSI and AVAT are two of four subscales of a Swedish modification of the Interview Schedule for Social Interaction (ISSI), which has been validated to be a reliable instrument for social structure assessment in a Swedish context [1,26]. For the 4 questions of AVSI, respondents were asked how many adults they interacted with and received support from in various ways, with 6 answer options ranging from no one to more than 15. The sum score of AVSI ranged from 4 to 24, with higher score indicating higher availability of social integration. For the 5 questions of AVAT, respondents were asked to what extent they agreed with positive statements about their availability to close relationships with adults and to support thereof. The answer for each question ranged from 1 “Do not agree at all” to 4 “Agree completely”, and the sum score thus varies between 5 and 20, with higher value indicating higher availability of attachment.

### Microsatellite genotyping

The collection of saliva for DNA using Oragene DNA sample collection kit (DNA Genotek Inc., Ottawa, Ontario, Canada) was described previously [27]. DNA was extracted from saliva samples using the Oragene Purifier. *AVPR1A* RS3 genotypes were determined using PCR primers: 5’-TCCTGTAGAGATGTAAGTGC-3’ (forward) and 5’-gtttcttTCTGGAAGAGACTTAGATGG-3’ (reverse) [15,21]. The amplification was made using AmpliTaq Gold (Applied Biosystems; Life Technologies, Carlsbad, CA, USA), 20–200 ng genomic DNA, 1 μM of each primer and 10% v/v 360 GC-Enhancer (Applied Biosystems) under the following conditions: 95°C for 5 min; 20 cycles of 95°C for 15 s, 65°C -0.5°C/cycle for 30 s, 72°C for 30 s, followed by 25 cycles of 95°C for 15 s, 55°C for 30 s, 72°C for 30 s and a final elongation step (72°C for 7 min). Eight negative controls were included in each 384-well plate. The sizes of the PCR products were determined using an ABI 3730 DNA Analyser (Applied Biosystems), the ABI GeneScan 500 LIZ size standard and the GeneMapper software version 4.0 (Applied Biosystems). Rate of successful genotyping was 85%. Of the 400 samples genotyped twice, 398 had identical allele lengths in the two runs.

### Statistical analysis

The microsatellite variants were categorized, based on the 334 bp ‘risk’ allele [18,21], in the short (S, ≤334 bp) or long (L, >334 bp) allele group as has been done previously [18] producing the genotype categories LL, SL or SS. Sociodemographic characteristics were initially tested for association to *AVPR1A* RS3 using ANOVA or Pearson’s *χ* ^*2*^ test. Thereafter, ANCOVAs were conducted on each of the outcome variables AVSI and AVAT, adjusting for the covariates age, gender and educational level; First, *AVPR1A* RS3 genotype was tested for association with AVSI and AVAT without consideration of childhood adversity. Second, the categorical CA and *AVPR* RS3 genotype were used as independent variables and also formed a two-way interaction term. Data were analyzed using the IBM SPSS Statistics 22.0 software (IBM Corporation, Somers, N.Y., USA). A two-sided *p*-value<0.05 was considered nominally statistically significant. Threshold for statistical significance was set to *p*<0.0125 (= 0.05/4, Bonferroni correction for 4 independent regression models: one model considering childhood adversity and one model not, each stratified for gender. Not more than four models were independent as correlation was high between AVSI and AVAT [*rho* = 0.49, *p*<0.0001] and between two-level and three-level CA [*rho* = 0.96, *p*<0.0001]).

## Results

### *AVPR1A* RS3 and social interaction without consideration of childhood adversity

The *AVPR1A* RS3 allele frequency distribution in the sample was comparable to previously published findings in Caucasians (Table 1)[21]. The AVSI and AVAT scores showed high correlation between wave 1 and wave 2 for the whole sample (*rho*_AVSI_ = 0.72 and *rho*_AVAT_ = 0.65, *p*<0.0005) and for those 549 individuals fulfilling the criteria for depression or anxiety in any wave (*rho*_AVSI_ = 0.69 and *rho*_AVAT_ = 0.66, *p*<0.0005). First we assessed the relationship between *AVPR1A* RS3 genetic variation and social interaction without consideration of childhood adversity. Sociodemographic characteristics of the participants are reported in Table 2. The three *AVPR1A* RS3 genotypes, SS, SL and LL, were dichotomized into SL+SS *vs* LL because the hippocampal *AVPR1A* expression level was similar between SL and SS groups [18]. When SS and SL groups were combined, LL carriers showed nominally lower AVAT scores also after taking age, gender and education into consideration (genotype: 17.8±2.5 vs. 17.5±2.7, *F* (1, 1848) = 4.92, *p* = 0.027; age: *p* = 0.027; gender: *p*<0.0005; education: *p* = 0.018) (Table 2). As gender had an effect on AVAT in the model, but not on AVSI (*p* = 0.26), gender specific analysis were done on AVAT. In males, AVAT differed nominally between S carriers and LL carriers (17.5±2.6 vs. 17.0±3.1, *F* (1, 743) = 3.97, *p* = 0.047), but not in females (18.0±2.4 vs. 17.8±2.4, *F* (1, 1105) = 1.43, *p* = 0.23) (Table 2). This suggested that there was a nominal main negative effect of genotype LL on AVAT score in males.

**Table 1:** Allele frequency distribution of *AVPR1A* RS3.

Allele	*n*	%
318	1	0.0003
320	44	0.012
324	3	0.0008
326	6	0.0016
328	7	0.0019
330	219	0.058
332	284	0.076
334	870	0.23
336	894	0.24
338	425	0.11
340	538	0.14
342	86	0.023
344	40	0.011
346	247	0.066
348	64	0.017
350	8	0.0021
352	6	0.0016

**Table 2:** Demographic characteristics, childhood adversity (CA) and social parameters across *AVPR1A* RS3 genotypes.

		*AVPR1A* RS3 genotypes (*n* = 1871)			*P* ^a^
		SS (*n* = 274)	SL (*n* = 886)	LL (*n* = 711)	
Gender, *n* (%)	Male (*n* = 748)	106 (39.0)	354 (40.2)	288 (40.9)	0.68
	Female (*n* = 1110)	166 (61.0)	527 (59.8)	417 (59.1)	
Age, mean ± SE		44.6±12.6	44.3±11.9	45.2±12.0	0.17
Education level, *n* (%)	<13 years (*n* = 1010)	158 (58.1)	460 (52.2)	392 (55.6)	0.39
	<12 years (*n* = 849)	114 (41.9)	422 (47.8)	313 (44.4)	
CA, mean ± SE		4.06 ±0.07	3.90±0.04	4.01±0.04	0.23
Two-levels, *n* (%)	non-CA (*n* = 888)	127 (46.7)	436 (49.4)	325 (46.1)	0.26
	CA(*n* = 971)	145 (53.3)	446 (50.6)	380 (53.9)	
Three-levels, *n* (%)	non-CA (*n* = 888)	127 (46.7)	436 (49.4)	325 (46.1)	0.50
	low-CA (*n* = 759)	112 (41.2)	352 (39.9)	295 (41.8)	
	high-CA (*n* = 212)	33 (12.1)	94 (10.7)	85 (12.1)	
AVSI^b^, mean ± SE		16.5±0.25	16.5±0.15	16.7±0.17	0.29^b^
AVAT^b^, mean ±SE	Males+females	17.7±0.14	17.8±0.09	17.5±0.11	0.027^b^
	Males (*n* = 742)	17.2±0.25	17.6±0.14	17.0±0.18	0.047^c^
	Females (*n* = 1105)	18.0±0.17	18.0±0.11	17.8±0.12	0.23^c^

AVSI: Availability of social integration.

AVAT: Availability of attachment.

CA: Childhood adversity.

^a^ Comparison between SS+SL and LL groups using ANOVA was performed for continuous variables and χ^2^ test for categorical variables.

^b^ ANCOVA adjusted for age, gender and education level.

^c^ ANCOVA adjusted for age and education level.

### *AVPR1A* RS3 and social interaction when childhood adversity was considered

**Fig 1:**
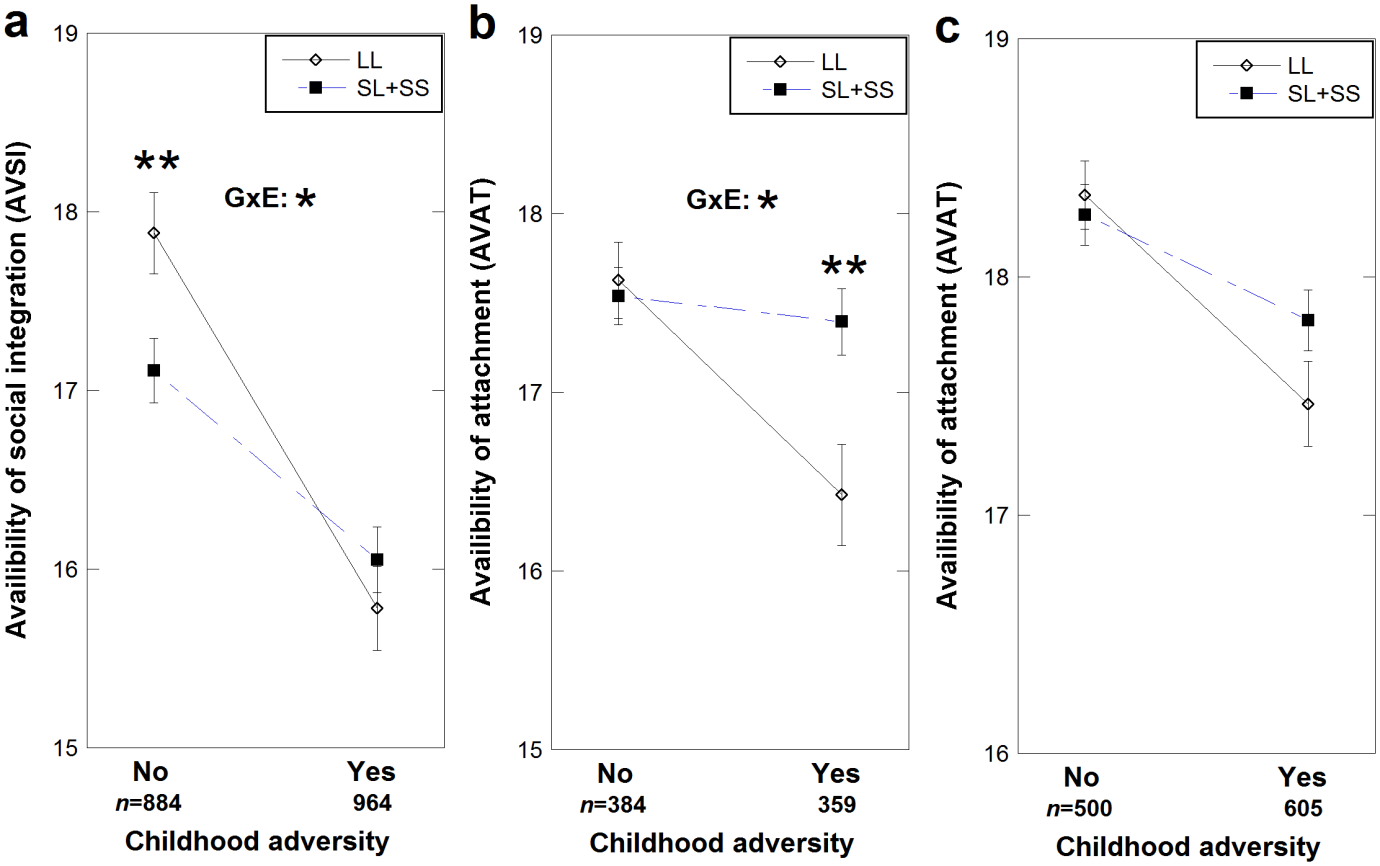
The interaction between *AVPR1A* RS3 and 2-level childhood adversity on the availability to social interaction. The interaction between *AVPR1A* RS3 and 2-level childhood adversity [CA, no or yes] on availability to social integration (AVSI) [a] and on availability to attachment (AVAT), in males [b] and females [c]). Dots indicate estimated marginal means and error bars represent one standard error of the mean. Open diamonds represent those homozygous for the long (L) allele group, filled squares represent the carriers of at least one short (S) allele. Statistical significance between genotypes in each childhood adversity group, as well as statistical significance of the *AVPR1A* * CA interaction (GXE) are indicated. * *P*<0.05, ** *P*<0.005.

Second, we assessed if there was an influence of *AVPR1A* RS3 on social interaction considering experience of adversity during childhood. The relationships for CA to AVSI and AVAT were significantly negative after controlling for age, gender and educational level: for AVSI (CA: *β*_*standardized*_ = -0.18, *p*<0.0005, age: *β*_*stand*_ = -0.005, *p* = 0.72, gender: *β*_*stand*_ = -0.002, *p* = 0.87, education: *β*_*stand*_ = -0.13, *p*<0.0005) and for AVAT (CA: *β*_*stand*_ = -0.13, *p*<0.0005, age: *β*_*stand*_ = -0.048, *p* = 0.001, gender: *β*_*stand*_ = -0.13, *p*<0.0005, education: *β*_*stand*_ = -0.046, *p*<0.0005). General linear models using CA introduced into the models as two-level categorical variables (CA as ‘no’ or ‘yes’), main effects of both CA and the interaction with genotype were significantly associated with AVSI and AVAT, as shown below, while there was a borderline signal (*p* = 0.050) for a main genotype effect only on AVAT, and not AVSI. The model was adjusted for age, gender and education, and a significant effect was found only for gender, and that on AVAT (*F* (1, 1848) = 31.1, *p*<0.0005) but not on AVSI (*F* (1, 1848) = 0.492, *p* = 0.48). Therefore, the final analysis was performed for males and females combined for AVSI, and genders separately for AVAT (Table 3). Thus, the occurrence of childhood adversity had a significant main negative effect on AVSI (*F* (1, 1848) = 45.9, *p*<0.0005, *Partial η* ^*2*^ = 0.024) and there was a significant interaction between CA and *AVPR* genotype on AVSI (*F* (1, 1848) = 8.14, *p* = 0.004, *Partial η* ^*2*^ = 0.004) (Fig 1a). The interaction effect was such that among participants reporting no CA, LL carriers had higher scores on AVSI (*p* = 0.002) compared to S carriers, however, the AVSI scores for LL carriers decreased more sharply following increased CA scores. Similar effects of interaction between CA and *AVPR* were seen in males on AVAT; A main negative effect of CA (*F* (1, 743) = 9.4, *p* = 0.002, *Partial η* ^*2*^ = 0.013) and a CA*AVPR effect (F (1, 743) = 6.5, p = 0.011, *Partial η* ^*2*^ = 0.009) with significant AVAT difference between genotypes in only those with childhood adversity (*p* = 0.003) (Fig 1b). In females however, only CA had an effect on AVAT (*F* (1, 1105) = 17.5, *p*<0.0005, *Partial η* ^*2*^ = 0.016) (Fig 1c). This suggested that LL carriers were more susceptible to childhood adversity for reducing social integration, and in males for reducing social attachment.

**Table 3:** ANCOVAs testing the effects of childhood adversity, *AVPR1A* RS3 genotypes (SS+SL *vs* LL) and their interaction on AVSI and AVAT.

		AVSI	
	*F*	*P*	*η2*
Model 1: *AVPR* genotype ^a^	2.30	0.13	0.001
CA (two-levels)	45.9	<0.0005	0.024
*AVPR*×CA (two-levels)	8.14	0.004	0.004
Model 2: *AVPR* genotype ^a^	0.32	0.57	<0.0005
CA (three-levels)	28.9	<0.0005	0.031
*AVPR*×CA (three-levels)	4.02	0.018	0.004
		AVAT	
	*F*	*P*	*η2*
Males			
Model 1: *AVPR* genotype ^a^	3.79	0.052	0.005
CA (two-levels)	9.39	0.002	0.013
AVPR*CA (two-levels)	6.51	0.011	0.009
Model 2: *AVPR* genotype ^a^	5.18	0.023	0.007
CA (three-levels)	5.17	0.006	0.014
AVPR*CA (three-levels)	3.19	0.042	0.009
Females			
Model 1: *AVPR* genotype ^a^	0.87	0.35	0.001
CA (two-levels)	17.5	<0.0005	0.016
AVPR*CA (two-levels)	2.34	0.13	0.002
Model 2: *AVPR* genotype ^a^	0.85	0.36	0.001
CA (three-levels)	9.61	<0.0005	0.017
AVPR*CA (three-levels)	1.44	0.24	0.003

*AVPR*: *AVPR1A* RS3.

AVSI: Availability of social integration.

AVAT: Availability of attachment.

CA: Childhood adversity.

ANCOVA adjusted for age, gender and education level.

^a^ Comparison between SS+SL and LL groups.

**Fig 2:**
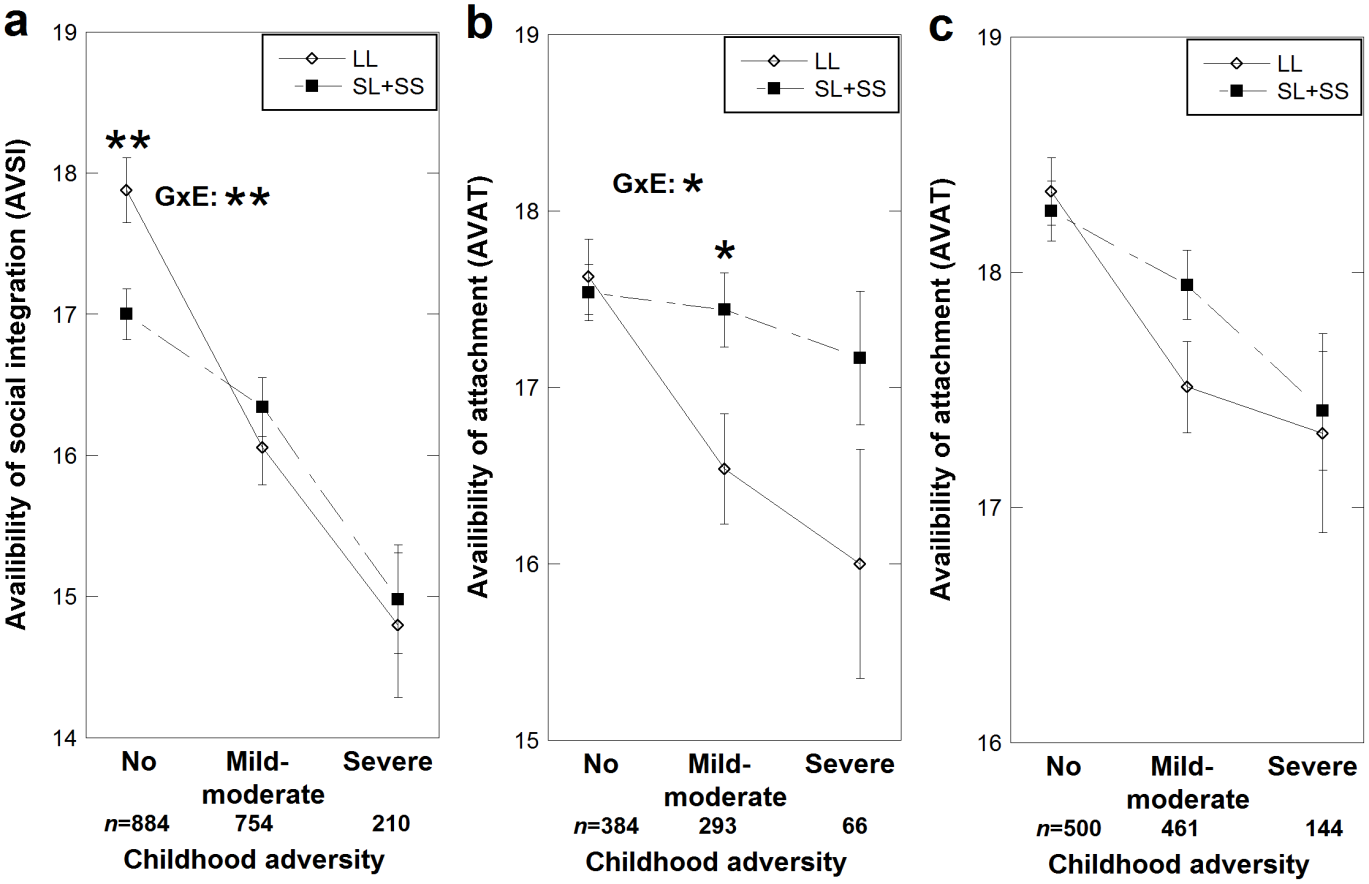
The interaction between *AVPR1A* RS3 and 3-level childhood adversity on the availability to social interaction. The interaction between *AVPR1A* RS3 and 3-level childhood adversity [CA, no, mild-moderate, severe] on availability to social integration (AVSI) [a] and on availability to attachment (AVAT), in males [b] and females [c]). Dots indicate estimated marginal means and error bars represent one standard error of the mean. Open diamonds represent those homozygous for the long (L) allele group, filled squares represent the carriers of at least one short (S) allele. Statistical significance between genotypes in specific childhood adversity group, as well as statistical significance of the *AVPR1A* * CA interaction (GXE) are indicated: * *P*<0.05, ** *P*<0.005.

To elucidate if the effect on social interaction was stronger for severe than mild-moderate level of childhood adversity (compared to no adversity), we separated those with severe levels of childhood adversity from those with mild-moderate; CA scores were coded as a three-level variable (non-CA, mild-moderate-CA, and high-CA) and introduced into the model. With regard to effects on AVSI, CA had a main effect (*F* (2, 1848) = 28.9, *p*<0.0005, *Partial η* ^*2*^ = 0.031) and also interacted with the *AVPR* genotype (*F* (2, 1848) = 4.02, *p* = 0.018, *Partial η* ^*2*^ = 0.004), while there was no effect of genotype, gender or age, but of education (*F* (1, 1848) = 29.5, *p*<0.0005, *Partial η* ^*2*^ = 0.016). The CA*AVPR interaction effect on AVSI was statistically significantly only between those that reported no and those that reported mild-moderate CA (*F* (1, 1638) = 7.07, *p* = 0.008, *Partial η* ^*2*^ = 0.004), whereas when CA increased further, the interaction disappeared (*p* = 0.89) and only the main effect of CA remained (*F* (1, 964) = 14.27, *p*<0.0005, *Partial η* ^*2*^ = 0.015) (Fig 2a). With regard to effects on AVAT, in males both CA and *AVPR* genotypes were predictors (*F* (2, 743) = 5.17, *p* = 0.006, *Partial η* ^*2*^ = 0.014 and *F* (1, 743) = 5.18, *p* = 0.023, *Partial η* ^*2*^ = 0.007, respectively), and a nominal CA*AVPR interaction was observed (*F* (2, 743) = 3.19, *p* = 0.042, *Partial η* ^*2*^ = 0.009), but no effect of age or education. Like for AVSI, the nominal interaction between CA and *AVPR* on AVAT in males was seen only for those exposed to no or mild-moderate CA (*F* (1, 677) = 5.17, *p* = 0.023, *Partial η* ^*2*^ = 0.008) (Fig 2b). When CA increased further, the scores of AVAT declined in similar trends among LL and S carriers (*F* (1, 359) = 0.096, *p* = 0.76) and only the main effect of genotype was nominally significant (*F* (1, 359) = 6.05, *p* = 0.014, *Partial η* ^*2*^ = 0.017) (Fig 2b). In females, no genotype or interaction effect was seen on AVAT (Fig 2c).

## Discussion

The main finding was that the individuals with longer *AVPR1A* RS3 alleles had a more negative effect of experienced childhood adversity on their current social interaction. With regard to social integration (AVSI), this relationship was found for both males and females, whereas for social attachment (AVAT) this genetic influence was found only among males.

More specifically, in males, those homozygous for the long alleles of *AVPR1A* RS3 (LL carriers) had nominally lower AVAT scores compared to short allele (S) carriers. And, this association was found to be restricted to those reporting experience of childhood adversity, whereas those carrying LL with no childhood adversity had similar AVAT score as S carriers. This suggests that among males, particularly those with LL genotype had an effect of childhood adversity on their social attachment in adulthood. A similar vulnerability to childhood adversity among LL carriers was found on social integration, but here both males and females were influenced. In fact, those LL had higher social integration, but only if they had not experienced childhood adversity. Severe experiences of childhood adversity is known to associate with reduced social interaction scores [28], which was shown also in our study. Our finding here that *AVPR1A* RS3 influences this dependence has to our knowledge not previously been reported. The higher social integration in LL carriers, that we found in those without childhood adversity, is in agreement with findings of better social cognition and higher altruism in those with long alleles [18,19].

It is notable that the statistical interaction between *AVPR1A* RS3 variants and childhood adversity occurred between no childhood adversity and mild-moderate childhood adversities, but disappeared when the adversities increased further. Then instead a main effect of childhood adversity explained the further reduction in AVSI. Thus, only AVSI was significantly reduced in those with severe childhood adversity compared to those with mild-moderate childhood adversity. On the other hand, in males *AVPR1A* RS3 had a main effect on AVAT regardless of severity of childhood adversity. These results suggest that, as for e.g. the *5-HTTLPR* [29], the effect of *AVPR1A* RS3 in part depends on early stressors. When early stress was reported as relatively weak, carriage of an S allele seemed to exert resilience against the stress’ effect on social integration. However, reported severe early stress sidelined the genetic effect on social integration. For social attachment in males however, severe early stress could not be shown to have a stronger effect than mild-moderate early stress, but again S carriers were more resilient against stress than those with LL genotype.

Male-specific *AVPR1A* RS3 effects were detected on AVAT. Studies across a broad range of species, including fish, amphibian, birds and mammals, demonstrated that the social behavioral effects of AVP are more prominent in males than females [7]. Likewise, Thompson et al.’s study on human samples reported gender specificity in the response to unfamiliar same-sex faces following intranasal administration of AVP [30,31]. Our result is also well in line with the previous observation that the association between *AVPR1A* RS3 334 bp ‘risk’ allele, belonging to the short allele group, and poor pair-bonding was found only in males [21].

A possible functionality of the *AVPR1A* RS3 repeat length has been suggested previously. LL carriers, in our study found to be particularly vulnerable to childhood adversity, were previously reported to have higher *AVPR1A* mRNA levels in human postmortem hippocampi [18]. The hippocampus, the amygdala and the surrounding temporal lobe are key in social behavior and social function [32,33]. Reduced volumes of hippocampus and amygdala have been reported among individuals with childhood trauma experiences [34–37]. In support of our finding, higher amygdala response to fearful faces was found in those carrying the *AVPR1A* RS3 long alleles [22]. Furthermore, intranasal administration of AVP increased autonomic responsiveness to threatening social stimuli and increased anxiety in humans [31]. Thus, we could assume that the genetic variants of *AVPR1A* RS3 may modulate the association between childhood adversity and social interaction through its expression in social function related brain structures, such as the hippocampus and the amygdala.

There are several limitations of this study. First, the cross-sectional design and retrospective data collection prevents causal inference. Longitudinal studies are essential to study causal relationships between genetic risk, early life adversity, and social development outcomes. Second, we used several self-reported items to assess social interaction. Our findings motivate replication efforts using more objective measures. Third, we had no information available on self-reported adequacy of social interaction, i.e. the wish for more social interaction. Fourth, data were from self-reported questionnaires and self-administered saliva collection. Hence, persons severely ill during data collection are likely under-represented in this study. Fifth, depression and anxiety diagnoses were overrepresented in the study group, approximately twice as high as in the whole PART population. In the subsample with depression and/or anxiety there was a main effect of *AVPR* RS3 on AVSI which was absent in the control subsample. However, the two subsamples showed similar directions of the CA*AVPR RS3 interaction (S1 Table). Sixth, recall bias in childhood adversity exists, but was limited since only 14% of the 2633 persons reporting childhood adversity in wave 1 did not do so in wave 2 (Forsell and Lundberg, unpublished). Also, childhood adversity was measured only by three questions, one being “friction in the family” which could mean different things to different persons. However, our findings of negative effect of childhood adversity on social interaction are consistent with previous studies [28]. Sixth, the rate of successful genotyping may be regarded as limited, 85%. However, the allele frequency distribution was similar to previous studies. Also, only 13.4% of the PART-individuals genotyped in the current study had a non-Swedish origin. Of those, the vast majority had a Nordic origin. Analysis including only the individuals with Swedish origin resulted in the same statistically significant findings, as those reported from the whole sample. The current Swedish population has no strong internal genetic borders [38] and especially the southern/middle parts of Sweden (from where the participants of this study are derived) are more genetically homogeneous [39].

Taken together, our results suggest that those homozygous for longer *AVPR1A* RS3 alleles, previously associated with higher hippocampal *AVPR1A* expression, had a more negative effect of experienced childhood adversity on their adulthood social interaction. To our knowledge, this is the first study reporting on the relationship between *AVPR1A* genetic variants and social interaction considering experiences of childhood adversities. The findings add to previous reports on higher acute vulnerability to stress in adults with long *AVPR1A* RS3 alleles and increased AVP levels [22,31].

## Supporting Information

S1 Table The effects of childhood adversity, *AVPR1A* RS3 genotypes (SS+SL *vs* LL) and their interaction on AVSI and AVAT in healthy controls and those with depression and/or anxiety. (DOCX)
