# Supplementary material for: Impact of Childhood Adversity and Vasopressin receptor 1a Variation on Social Interaction in Adulthood: A Cross-Sectional Study
Source: PLoS One. 2015 Aug 21;10(8):e0136436. doi: 10.1371/journal.pone.0136436 (PMC4546684; doi:10.1371/journal.pone.0136436)
Supplement: S1 Table — (DOCX) [file pone.0136436.s001.docx]

**Supporting information S1**.

**S1 Table**. The effects of childhood adversity, *AVPR1A* RS3 genotypes (SS+SL *vs* LL) and their interaction on AVSI and AVAT in healthy controls and those with depression and/or anxiety.

|  | AVSI | | | | | | | | |
| --- | --- | --- | --- | --- | --- | --- | --- | --- | --- |
|  | Healthy controls (n=1303) | | | |  | Depression and/or anxiety (*n*=539) | | | |
|  | *F* | *P* | *η2* | *B* |  | *F* | *P* | *η2* | *B* |
| *AVPR* genotype ^a^ | 0.014 | 0.91 | 0 | 0.49 |  | 9.10 | 0.003 | 0.017 | -0.17 |
| CA | 7.39 | 0.007 | 0.006 | 1.09 |  | 24.06 | ＜0.0005 | 0.043 | 3.04 |
| AVPR×CA | 4.08 | 0.044 | 0.003 | -0.92 |  | 6.72 | 0.010 | 0.012 | -2.10 |
|  |  |  |  |  |  |  |  |  |  |
|  | AVAT | | | | | | | | |
|  | Healthy controls | | | |  | Depression and/or anxiety | | | |
|  | *F* | *P* | *η2* | *B* |  | *F* | *P* | *η2* | *B* |
| Males | *n*=578 |  |  |  |  | *n*=163 |  |  |  |
| *AVPR* genotype ^a^ | 3.90 | 0.049 | 0.007 | 0.81 |  | 0.55 | 0.46 | 0.004 | 1.28 |
| CA | 0.33 | 0.57 | 0.001 | 0.31 |  | 6.77 | 0.010 | 0.041 | 2.51 |
| *AVPR*×CA | 4.56 | 0.033 | 0.008 | -0.84 |  | 1.62 | 0.20 | 0.010 | -1.62 |
|  |  |  |  |  |  |  |  |  |  |
| Females | *n*=725 |  |  |  |  | *n*=376 |  |  |  |
| *AVPR* genotype ^a^ | 2.25 | 0.13 | 0.003 | 0.23 |  | 0.064 | 0.80 | 0 | 0.46 |
| CA | 5.06 | 0.025 | 0.007 | 0.33 |  | 6.25 | 0.013 | 0.017 | 1.38 |
| *AVPR*×CA | 0.044 | 0.83 | 0 | -0.056 |  | 2.73 | 0.100 | 0.007 | -1.10 |

AVSI: Availability of social integration.

AVAT: Availability of attachment.

CA: Childhood adversity, two-level

*B*: unstandardized beta

ANCOVA adjusted for age, gender and education level.

^a^ Comparison between SS+SL and LL groups (LL being reference).
